# Chromium propionate supplementation to energy- and protein-reduced diets reduces feed consumption but improves feed conversion ratio of yellow-feathered male broilers in the early period and improves meat quality

**DOI:** 10.1016/j.psj.2023.103260

**Published:** 2023-11-07

**Authors:** Qianqian Zhang, Hongtao Zhang, Yukun Jiang, Jianping Wang, De Wu, Caimei Wu, Lianqiang Che, Yan Lin, Yong Zhuo, Zheng Luo, Kangkang Nie, Jian Li

**Affiliations:** ⁎ Department of Animal Resources and Science, Dankook University, Cheonan 31116, South Korea; † Key Laboratory of Animal Disease-Resistant Nutrition of Sichuan Province, Institute of Animal Nutrition, Sichuan Agricultural University, Chengdu 611130, China; ‡ Kemin (China) Technologies Co., Ltd., Zhuhai, China

**Keywords:** carcass trait, chromium propionate, growth performance, nutrient density, nutrient transporter gene

## Abstract

Growth performance and carcass traits may be retarded by low nutrient density diets. Organic chromium propionate (**CrProp**) can improve growth, carcass traits, and meat quality in farmed lambs, white broilers, and fish. Limited data regarding CrProp's impacts on yellow-feathered broilers are available. Eight hundred yellow-feathered male broilers (1-day old) were randomly allocated to 4 dietary groups and reared for 56 d. The trial was a 2 (dietary nutrient density) ×2 (CrProp) factorial arrangement with 4 diets: regular nutrient diet and low nutrient density (**LND**, reduction in metabolizable energy by 81 kcal and crude protein by 0.43%) diet supplemented with or without 200 mg/kg CrProp. Broilers were euthanized at d 56 after blood collection. The results indicated that the LND diet led to greater average daily feed intake (**ADFI**) from d 1 to 42 and feed conversion ratio (**FCR**) from d 22 to 42 (*P* < 0.05). Supplementation of CrProp improved body weight (**BW**) from d 1 to 56, average daily gain (**ADG**), and FCR during d 1 to 42 but reduced ADFI during d 1 to 21, as well as lowered abdominal fat percentage (*P* < 0.05). Supplementation with CrProp to regular and LND diets reduced ADFI but improved FCR from d 1 to 21 (*P* < 0.05). The LND diet lowered total antioxidant capacity (**T-AOC**) concentration and total superoxide dismutase (**T-SOD**) activity in the jejunal mucosa. CrProp elevated T-AOC levels and glutathione peroxidase activity (**GSH-Px**, *P* < 0.05). Dietary CrProp upregulated (*P* < 0.05) the expression of fatty acid transporter (**FABP1**) gene and peptide transporter (**Pept1**) gene. CrProp administration increased jejunal FABP1 expression and lowered cooking loss of breast meat (*P* < 0.05) in the LND group while reducing shear force (*P* = 0.009) of broilers treated by regular diet. In summary, CrProp administration to the LND diet can improve growth performance in the starter period and meat quality on d 56, possibly through upregulated nutrient transporter gene expression in the jejunum and enhanced antioxidant capability.

## INTRODUCTION

Despite the genetic potential, energy and protein sources are essential nutrients for optimal growth performance. However, they account for most of the feed costs and total production expenditure (El-Senousey et al., 2019; Attia et al., 2021). In facing the rising grain prices and the requirements of reducing pollutant emissions such as NH_3_, new insight into modulating formulations has been gained by lowering dietary metabolic energy (**ME**) and crude protein (**CP**). Consequently, reducing ME by 143 kcal/kg and CP by 1% (the constant ME:CP ratio) limited Hubbard broilers’ growth rates and increased feed conversion ratios (**FCR**) (Kamran et al., 2008). Other low nutrient density diets with constant ME: CP ratios resulted in lighter body weight (**BW**) and increased FCR in commercial broilers (Paraskeuas et al., 2016; Attia et al., 2022). Carcass yields and meat quality are crucial economic parameters. However, the utilization of high nutrient density diet to obtain heavier BW and more meat yield, results in more abdominal fat deposition (Ahmadi-Sefat et al., 2022; Alagawany et al., 2022) and negative impacts on meat quality such as color, tenderness, and pH (Niu et al., 2009; Zhao et al., 2012; Zhu et al., 2012). These quality traits affect consumers’ sensory and purchase desires.

Chinese yellow-feathered chickens have been favored by consumers in recent years for their excellent tasting experience, and they exceeded white feather broilers in production during 2014 to 2019 in China (Wang et al., 2017). However, yellow-feathered broilers have higher FCR ranging from 1.8 to 2.6 and slower growth rates. Because of this, they have a more extended feeding period than commercial broilers (Tang et al., 2021). Furthermore, they deposit fat in the abdomen faster than white-feathered broilers (Weng et al., 2022). For poultry enterprises, reducing nutrient density to control feeding costs is a sustainable development strategy with the premise of unaffecting growth rates.

Chromium (**Cr**), a micronutrient, may help achieve this. Cr is a vital glucose tolerance factor (**GTF**) component that promotes insulin binding with cell membrane receptors, further stimulating glucose absorption for glycogen synthesis (Brooks et al., 2016; Zhang et al., 2022a). Data from animal studies demonstrate that organic Cr is efficiently absorbed 20- to 30-folds greater in the gastrointestinal tract due to its higher water-solubility than inorganic Cr. Chromium is present in all tissues with high concentrations in the liver and kidney and low levels in the muscles of juvenile mud crabs (Zhang et al., 2022b). Trivalent chromium ions (**Cr^3+^**), such as chromium yeast, chromium pyridine, chromium methionine, and chromium propionate, are commonly used in laboratory and farmed animals (Kroliczewska et al., 2004) to investigate its effect. In several studies, organic Cr^3+^ compounds have shown to improve the average daily gain (**ADG**) and FCR in commercial broilers and quails (Sahin et al., 2010; Arif et al., 2019; Hayat et al., 2020; Huang et al., 2020; Van Hoeck et al., 2020), as well as carcass and breast meat yields (Rajalekshmi et al., 2014; Van Hoeck et al., 2020). Other studies report that Cr^3+^ modulates fat metabolism, such as diminished fat deposition in lambs and broilers but increases n-3 polyunsaturated fatty acids in the muscles of broilers and pigs (Toghyani et al., 2012; Moreno-Camarena et al., 2015; Jin et al., 2018; Han et al., 2021). Moreover, Cr is a recognized antioxidant associated with suppressed corticosterone, as evidenced by decreased lipid peroxidation in muscle and hepatopancreas in fish and broilers (Untea et al., 2019; Zhang et al., 2022b), corresponding to the requirement of adding Cr in diets (NRC, 1997). A high dose of Cr, however, can disrupt the redox balance, damage DNA, and induce cancers (Hayat et al., 2020; Monga et al., 2022). In China, chromium propionate (**CrProp**) is the only organic Cr source approved for broiler usage in 2022, with a maximum dosage of 0.2 mg Cr/kg in the feed.

Considering the effects of Cr^3+^ and the food cost-saving needs in intensive yellow-feathered broiler production system, we hypothesize that i) CrProp supplementation to a low nutrient density diet (different MP:CP ratios) could achieve comparable growth performance of yellow-feathered broilers with those fed the regular diet; ii) CrProp supplementation may improve the growth performance of broilers fed a regular diet. In the present study, yellow-feathered broilers fed with a low nutrient diet, or a regular diet supplemented with or without CrProp, were used to validate their effects on growth performance, carcass traits, meat quality, intestinal antioxidant activity, and nutrient transporter gene expressions.

## MATERIALS AND METHODS

Broilers were raised at the poultry farm in Ya'an, Sichuan, China. All feeding, vaccination programs, and sampling protocols were reviewed and approved by Sichuan Agricultural University's Animal Care and Use Committee (protocol No:20220520).

### CrProp Product

The CrProp [Kemin (China) Technologies Co., Ltd., Zhuhai, China] contains 0.09 to 0.10% Cr^3+^ and less than 5% water, with the medical stone as carrier.

### Trial Design and Diet

Eight hundred yellow-feathered male chicks at 1-day old were randomly allocated to 4 dietary treatment groups with an initial BW of 41.4 ± 0.1 g and reared until 56 d of age. Each treatment contained 200 chicks in 8 replicate cages of 25 chicks each. This trial was a 2 × 2 factorial design: regular diet, regular diet plus 200 mg/kg CrProp (0.2 mg Cr^3+^/kg), low nutrient density diet (**LND**), and LND diet plus 200 mg/kg CrProp. The regular diet (corn-soybean meal feed) was formulated according to the Chinese Feeding Standard for native chickens (NY/T3645-2020) in 3 phases: starter period (d 1–21), grower period (d 22–42), and finisher period (d 43–56). The LND diet was formulated by decreasing 1% soybean oil (reduction in ME, 81 kcal/kg) and 0.76% soybean meal (reduction in CP, 0.43%) from the regular diet. Between the 2 diets, ME: CP ratios did not match. Table 1 displays the detailed dietary compositions.

**Table 1:** Ingredients and composition of diets.

Ingredients	Regular diet			Low nutrient density diet		
	D 1–21	D 22–42	D 43–56	D 1–21	D 22–42	D 43–56
Corn	56.29	59.22	59.79	56.29	59.22	59.79
Soybean meal, 43%	29	30	28	28	29	27
Corn protein flour, 58%	8.2	4	2	8.2	4	2
Soybean oil	2	3	6.9	1.34	2.34	6.24
Calcium hydrogen phosphate	2	1.6	1.1	2	1.6	1.1
Limestone	1.1	1.1	1.2	1.1	1.1	1.2
Unite bran	-	-	-	1.66	1.66	1.66
Broiler vitamins^1^	0.03	0.03	0.03	0.03	0.03	0.03
Broiler minerals^2^	0.12	0.12	0.1	0.12	0.12	0.1
Nonstarch polysaccharase	0.02	0.02	0.02	0.02	0.02	0.02
Phytase	0.02	0.02	0.02	0.02	0.02	0.02
Choline chloride, 50%	0.1	0.1	0.08	0.1	0.1	0.08
L-Lysine monohydrochloride, 78.8%	0.5	0.17	0.19	0.5	0.17	0.19
L-Methionine, 99%	0.16	0.16	0.15	0.16	0.16	0.15
NaHCO_3_	0.26	0.26	0.24	0.26	0.26	0.24
NaCl	0.2	0.2	0.18	0.2	0.2	0.18
Total	100	100	100	100	100	100
Calculated composition						
Metabolic metabolites (kcal/kg)	2953.29	3007.93	3255.53	2872.21	2926.85	3174.45
Crude protein (%)	22.01	20.04	18.07	21.58	19.61	17.64
Crude fat (%)	4.59	5.66	9.50	3.92	4.99	8.83
Ca (%)	0.97	0.88	0.80	0.96	0.88	0.80
Available phosphorus (%)	0.45	0.38	0.30	0.45	0.38	0.30
Lysine (%)	1.19	1.00	0.95	1.17	0.98	0.92
Methionine (%)	0.54	0.50	0.45	0.54	0.49	0.44
Analyzed Cr, mg/kg	0.62	0.58	0.60	0.63	0.58	0.61

1 Provided per kg of diet: vitamin A, 12,000 IU; vitamin D3, 3,000 IU, vitamin E, 30 IU; vitamin K3, 4.8 mg; vitamin B1, 3 mg, vitamin B2, 9.6 mg; vitamin B6, 6 mg; D-biotin, 1.67 mg, D-pantothenic acid,18 mg; folic acid, 1.5 mg; nicotinamide, 600 mg.

2 Provided per kg of diet: 8 mg/kg Cu (as CuSO_4_·H_2_O); 80 mg/kg Fe (FeSO_4_·H_2_O); 100 mg/kg Mn (MnSO_4_·H_2_O); 0.7 mg/kg I (KI); 0.35 mg/kg Se (Na_2_SeO_3_).

### Housing and Management

All broilers were housed in a 2-layer stainless steel cage with the same size cage (300 cm × 80 cm × 60 cm). In the first week, the room temperature was kept at 29°C ± 2°C by heat lamps, then declined by 2°C each week until it reached 20°C. Throughout the entire period, the light was turned on for 23 h and off for 1 h. Relative humidity was 55 to 60%. Broilers had free access to tap water and feed through 6 nipple drinkers and 2 feed troughs in each cage.

### Measurements

The broilers were weighed by cage after starvation for 6 h at d 21, 42, and 56, feed consumption was recorded and used to calculate the ADG, average daily feed intake (**ADFI**), and FCR. Broilers with deformed legs that cannot eat and drink were culled as a percentage of mortality during the feeding period. The broilers with BW far below the averages were excluded from the analysis. The detailed incidence mortality information can be found in Table 2.

**Table 2:** Mortality records in each treatment.

Diagnosis, broiler	Regular	Regular + CrProp	LND	LND + CrProp
Leg abnormalities	1	3	4	2
Poor performance	1	0	1	4
Dead number, per treatment	12	12	11	7
%, per treatment^1^	7.0%	7.5%	8.0%	6.5%

1 Total regarded dead broilers each treatment / 200 broilers per treatment.

### Determination of Dietary CrProp

Dietary Cr was analyzed by wet ashing the feed sample with trace metal grade HNO_3_ (Trace Metal Grade, Fisher Scientific, Raleigh, NC) using a hot plate digestion procedure (Brooks et al., 2016). Cr was measured using flameless atomic absorption spectrophotometry (Shimadzu, Model AA-7000, Kyoto, Japan).

### Carcass Sampling

At d 56, 1 broiler per cage (whose BW was closest to the average BW of the treatment) was selected after checking live BW. Eight broilers per treatment were euthanized and bleeding for 5 min. Feather removal was processed after submerging in hot water at 60°C for 2 min. The carcass weight was recorded after bleeding and feather removal. The trachea, esophagus, crop, intestines, spleen, pancreas, gut, bursa of Fabricius, head, feet, liver, gizzard, spleen, and abdominal fat were removed by skilled personnel. Weights were determined for skinned breast meat (2 sides) and leg meat (2 sides; thigh + calf) without bones. The semieviscerated yield and eviscerated yield were the percentage of semieviscerated or eviscerated carcass weight relative to live BW. Breast meat and thigh meat yield were the percentage of breast meat or thigh meat weight relative to eviscerated weight, and abdominal fat yield was the percentage of abdominal fat weight relative to carcass weight.

### Meat and Tissue Sampling

One broiler randomly selected from each cage was used for meat and tissue sampling. Blood sample was collected into a 10 mL vacuum tube via wing vein. Serum was obtained by centrifuging blood sample at 4,000 × *g*, 4°C for 10 min and stored at −20°C. The broiler was euthanized after blood sampling. The skinned left breast meat was removed entirely for meat quality assessment. The right lobe liver tissues were sampled, quickly frozen in liquid nitrogen, and kept at −80°C. Jejunal tissues (midpoint between the pancreatic loop and Meckel's diverticulum) were longitudinally cut and washed in ice-cold normal saline (0.9%), then kept at −80°C. The mucous membranes were scraped from the remaining jejunal tissues and stored at −80°C for further analysis.

### Evaluation of Meat Quality

At 45 min and 24 h postmortem, muscle pH was measured using a pH probe (Testo 205, AG, Lenzkirch, Germany). The pH value was the average of 3 readings after the probe was randomly inserted into the muscle (about 2 cm depth). The meat color: L* (lightness), a* (redness), and b* (yellowness) was determined using a Minolta reflectance colorimeter (CR-300, Konica Minolta Sensing, Osaka, Japan) by placing the colorimeter on the muscle cross-sectional surfaces, with 3 random points of breast meat after 45 min postmortem. The chroma meter was calibrated by L* = 100.00, a* = 0.32, b* = 0.33). About 35 g of diced meat was enveloped in a sample bag, hung in cold storage at 4°C for 24 h, and then weighed again. The drip loss was the percentage of the weight difference between the initial and second weight relative to the initial weight. As previously described by Wen et al. (2017), after being chilled for 24 h, the muscle was wiped off surface moisture, weighed (about 100 g), and sealed in plastic wrap. Then, they were heated in a water bath at 70°C for 30 min to determine the loss after cooking. Shear force measurement was performed on the cooked meat. Each sample was sheared perpendicular to the grain of the muscle fiber using a texture analyzer (TA-XT plus, Stable Micro Systems, UK). The maximum force measured to cut the cores was expressed in kilograms.

### Biochemical Indices

The insulin activity was immediately measured using an ELISA kit (Catalog No H203) with a determination limit of 0.5 to 200 mIU/mL and intra-assay coefficient of variation under 9%. Corticosterone concentration was assayed using the corresponding ELISA kit (Catalog No: H094) with intra-assay coefficient of variation under 7.5% and a detection limit of 2 to 600 ng/mL. The 2 kits were purchased from Nanjing Jiancheng Bioengineering Institute, Nanjing, China.

### Lipid Peroxidation and Antioxidant Enzymes in the Jejunal Mucosa

Oxidation products and antioxidant enzyme activity evaluate the jejunal antioxidant defense system in the mucosa. Thus, we determined total antioxidant capacity (T-AOC; Catalog No. A015-2), malondialdehyde (**MDA**; Catalog No. A003-1), superoxide dismutase (**SOD**; Catalog No. A001-1), and glutathione peroxidase (**GSH-Px**; Catalog No. A005-1) using commercial kits purchased from Nanjing Jiancheng Co. Ltd. (Nanjing, China). Frozen jejunal mucosa sample was ground into powder in a mortar filled with liquid nitrogen and stored at −80°C. One gram of powder was homogenized with 9 × volume of phosphate buffer for 30 s, then certificated at 2,500 × *g*, 4°C for 10 min. As described by Wan et al. (2016), a pink substance was formed by the condensation reaction of MDA and thiobarbituric acids, which can be detected with a microplate at 532 to 535 nm (SpectraMax 190, Molecular Devices, Sunnyvale, CA). T-AOC was assayed by colorimetric analysis at 520 nm (Zhang et al., 2013). Briefly, antioxidants reduce Fe^3+^ to Fe^2+^, which compounds with green phenanthroline. As described by Jia et al. (2011), 1 unit of SOD activity is defined as the amount of SOD required to inhibit 50% of the xanthine oxidase system reactions at 450 nm. GSH-Px represents the speed at which reduced glutathione is oxidized by H_2_O_2_ to oxidized glutathione. Each parameter was assayed in duplicate, following the instructions strictly. The protein concentration in the supernatant was measured using the BCA protein assay kit (Beyotime Biotechnology; Shanghai, China).

### Quantitative Real-Time PCR

The expressions of fatty acid transporter gene (**FABP1**), Y+L amino acid transporter 1 gene (y+, LAT1), glucose transporter gene (**GLUT2**), peptide transporter 1 gene (**Pept1**), sodium-dependent glucose transporter gene (**SGLT1**), cluster of differentiation 36 gene (**CD36**), cationic amino acid transporter 1 gene (**CAT1**), and glucose transporter 2 gene (**GLUT2**) in jejunal tissues and insulin-like growth factor gene (**IGF1**) and growth hormone gene (**GH**) in hepatic tissues were determined by quantitative real-time PCR. Tissue RNA was extracted with trizol reagent (Vazyme, Nanjing, China), and the quantity and quality of RNA were checked using a NanoDrop 2000 spectrophotometer (Thermo Fisher Scientific, Inc., Wilmington, DE). The extracted RNA was reverse transcribed into cDNA using HiScript-RT SuperMix kit (Vazyme, Nanjing, China) with a reaction system of 20 uL. Primers in Table 3 were designed based on NCBI sequences. The 20 uL reaction volume included 10 uL SYBR qPCR Green Master Mix (Vazyme, Nanjing, China), 0.4 uL forward primer, 0.4 uL reverse primer, 1 uL cDNA, and 8.2 uL ddH_2_O. All reagents were added to 384 microwell plates, and the sample was assayed in triplicate. According to Applied Biosystems QuantStudio5 (Thermo Fisher), the PCR reaction procedures included predenaturation (95°C for 30 s), followed by 40 cycles (95°C for 10 s and 60°C for 30 s) and melting curve period (95°C for 15 s, 60°C for 60 s, and 95°C for 15 s). The calculation of mRNA expression used the 2^‐ ΔΔCt^ method (Livak and Schmittgen, 2001) after standardization by β-actin.

**Table 3:** Primer sequences used in quantitative real-time PCR.

Genes	Primer sequence	GenBank accession no.
SGLT1	Forward: CCACCGCCATAAGGATCAACA Reverse: GTTGGTTGAGTACATAGCCCATAC	NM_001293240.2
FABP1	Forward: TGGGGAAGAGTGTGAGATG Reverse: ATTGTATGGGTGATGGTGTCT	NM _204192.4
GLUT-2	Forward: AAAGCAAAATGCAGGCGGAG Reverse: GTGCTTCTATCACCTTCTGCG	NM_207178.2
y+LAT1	Forward: ACTTCACAACCCTGTCCACG Reverse: CAACGAAGAACAGCCGGGAA	NM_001030579.3
CAT1	Forward: AACTGGGTTTCTGCCAGAGG Reverse: ACCCATGATGCAGGTGGAG	NM_001145490.1
CD36	Forward: ATGTTGTCGCCACTGTCTTG Reverse: GGCTATCAGGTTCTGCCTGT	NM_205512.1
PepT1	Forward: TAAGAGTAAGGGCCGATCAGTG Reverse: ACGTGTGGTAGATGGCTGTAG	NM_204365.1
Beta-actin	Forward: CTGTGATGAAACAAAACCCA Reverse: GACTGCTGCTGACACCTTC	L08165.1
IGF	Forward: TCCAGCAGTAGACGCTTACAC Reverse: GAGCACAGTACATCTCCAGCC	NM_001004384.3
GH	Forward: AGCGATGACCAGGCAGAGGA Reverse: CCAGCTCAGTTTTGGACCCG	NM_204359.2

SGLT1, sodium-dependent glucose transporter 1; FABP1, fatty acid-binding protein; GLUT2, glucose transporter 2; y+, LAT1, Y + L amino acid transporter 1; CAT1, cationic amino acid transporter 1; CD36, cluster of differentiation 36; PepT1, peptide-transporters 1; IGF, insulin-like growth hormone; GH, growth factor.

### Statistical Analysis

The data were analyzed using the 2 × 2 GLIMMIX procedure (SAS 9.4, Institute Inc., Cary, NC), with diet, CrProp, and their interaction as fixed effects. Except for gene expression with 6 replications in each treatment, 8 replication values were used for SAS analysis for all other parameters. When the interactive effects were significant, means were compared using Tukey's post hoc test among the 4 treatments. Results were expressed as means ± pooled SEM from least-square means, with *P* < 0.05 declared significant. Data were visualized using GraphPad 9.0.

## RESULTS

### Growth Performance and Serum Parameters

The mortality among treatments ranged from 7.0, 7.5, 8.0, and 6.5% (Table 2), and there was no significant difference. Table 4 presents the impacts of dietary nutrient density and CrProp on the growth performance of yellow feather broilers. The LND diet promoted more ADFI during d 1 to 21 and d 22 to 42 (*P* < 0.05) and increased the FCR of broilers from d 22 to 42 (*P* = 0.004) compared to the regular diet. Dietary CrProp improved overall BW, ADG, and FCR in the first 42 d, and ADFI in the first 21 d (*P* < 0.05), relative to the no-CrProp group. The interactive effects between CrProp and LND diet reduced ADFI from d 1 to 21 (*P* = 0.012) but improved the FCR.

**Table 4:** Growth performance responds to dietary nutrient density and CrProp.

Items		Body weight, g/broiler				ADG, g/broiler			ADFI, g/broiler			Feed conversion ratio		
Nutrient density	CrProp	D 1	D 21	D 42	D 56	D 1–21	D 22–42	D 43–56	D 1–21	D 22–42	D 43–56	D 1–21	D 22–42	D 43–56
Normal	−	41.44	510.87	1455.11	2182.41	22.35	44.51	52.63	35.68^b^	95.25	136.71	1.596^b^	2.140	2.671
	+	41.35	524.91	1491.88	2226.18	23.03	45.55	53.19	35.78^b^	95.85	138.83	1.555^b^^c^	2.105	2.536
Low	−	41.43	513.73	1445.59	2171.96	22.49	44.83	51.20	37.46^a^	99.55	135.83	1.666^a^	2.221	2.604
	+	41.41	528.98	1481.41	2258.78	23.22	45.85	54.78	35.74^b^	98.13	135.85	1.539^c^	2.140	2.533
SEM		0.054	4.10	6.43	20.05	0.20	0.32	1.46	0.34	0.89	2.72	0.015	0.019	0.058
Nutrient density														
Normal		41.42	517.88	1463.44	2204.13	22.69	45.04	52.91	35.74^b^	95.54^b^	135.14	1.576	2.122^b^	2.568
Low		41.39	521.25	1473.44	2215.31	22.85	45.34	52.99	36.60^a^	98.95^a^	137.77	1.603	2.180^a^	2.603
SEM		0.036	2.39	5.14	16.05	0.11	0.25	1.21	0.33	0.55	2.15	0.013	0.011	0.036
CrProp														
−		41.43	512.25^b^	1450.38^b^	2177.06^b^	23.22^b^	44.67^b^	51.90	36.57	96.99	136.63	1.631^a^	2.181^a^	2.637
+		41.38	526.88^a^	1486.50^a^	2242.38^a^	22.43^a^	45.71^a^	53.99	35.77	97.41	136.28	1.547^b^	2.122^b^	2.534
SEM		0.045	3.25	5.27	14.10	0.16	0.27	1.08	0.29	0.71	2.22	0.012	0.013	0.036
*P* value														
Diet		0.649	0.406	0.131	0.585	0.416	0.345	0.958	0.016	0.001	0.339	0.081	0.004	0.553
CrProp		0.366	0.001	<0.001	0.003	0.001	0.004	0.168	0.023	0.650	0.903	<0.001	0.004	0.087
Interaction		0.496	0.884	0.942	0.292	0.890	0.985	0.313	0.012	0.266	0.517	0.008	0.228	0.585

Values are represented as means ± SEM (*n* = 8).

a–c Mean values without common superscript differ significantly (*P* < 0.05).

**Figure 1:**
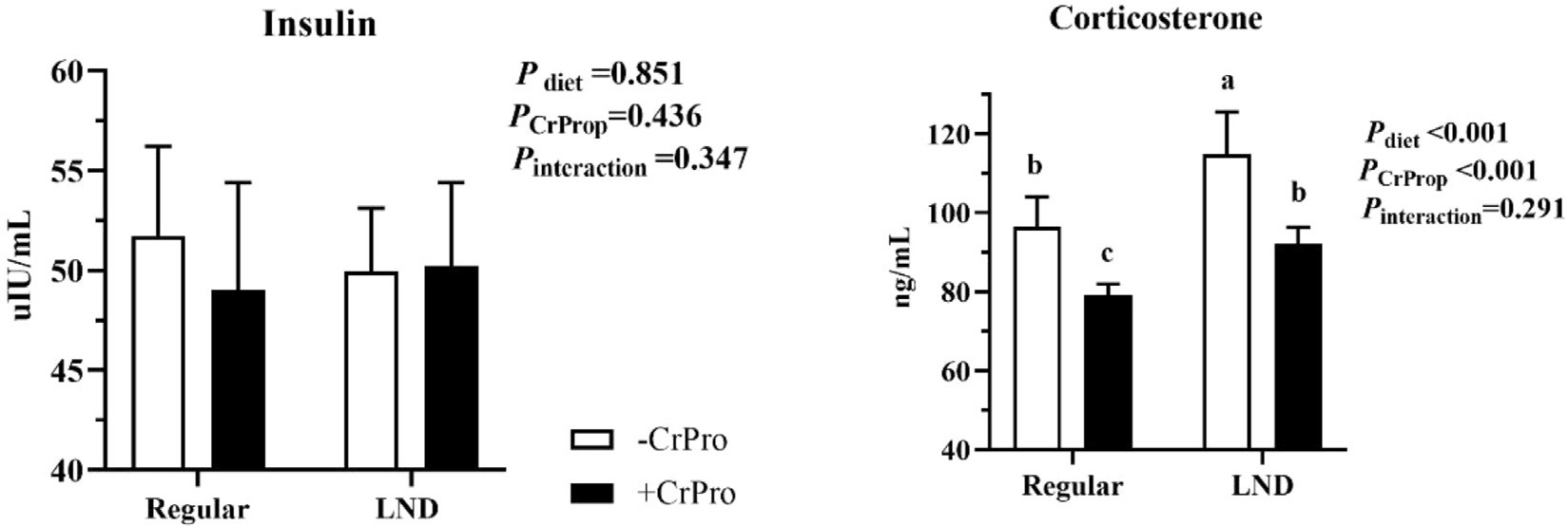
Serum parameters respond to dietary nutrient density and CrProp. Values are represented as means ± SEM (*n* = 8). ^a,b^Mean values without common superscript differ significantly (*P* < 0.05).

As presented in Figure 1, the LND diet led to higher serum corticosterone concentration (*P* < 0.01) than the regular diet. While CrProp supplementation reduced corticosterone (*P* < 0.01) at d 56, relative to the no-CrProp diet. Insulin activity was neither affected (*P* > 0.05) by nutritional density, CrProp, nor their interaction at d 56.

### Carcass Traits

The carcass weight, eviscerated yield, semieviscerated yield, and breast and thigh muscle yield were not affected (*P* > 0.05) by treatments (Table 5; *P* > 0.05). The abdominal fat yield was reduced by CrProp supplementation (*P* < 0.01).

**Table 5:** Carcass parameters responds to dietary nutrient density and CrProp on d 56.

Items		Live BW, g	Carcass weight, g	Eviscerated yield, %	Semieviscerated yield, %	Breast yield, %	Thigh yield, %	Abdominal fat, %
Nutrient density	CrProp							

Normal	−	2247.50	2056.25	82.79	67.48	30.51	40.32	2.54
	+	2250.00	2075.00	83.88	68.09	28.69	40.06	2.36
Low	−	2220.00	2036.25	83.91	67.22	27.99	41.02	2.47
	+	2245.00	2058.75	83.47	68.31	28.53	39.15	2.32
SEM		18.77	19.16	0.40	0.48	0.99	0.65	0.03
Nutrient density								
Normal		2248.75	2065.63	83.34	67.78	29.60	40.19	2.42
Low		2240.88	2050.63	83.68	67.76	28.25	40.08	2.45
SEM		11.29	11.47	0.32	0.33	0.70	0.46	0.02
CrProp								
−		2242.13	2049.38	83.68	67.35	29.25	40.67	2.50^a^
+		2247.50	2066.88	83.35	68.20	28.61	39.60	2.37^b^
SEM		12.97	13.33	0.30	0.34	0.70	0.46	0.02
*P* value								
Diet		0.394	0.352	0.383	0.964	0.185	0.876	0.463
CrProp		0.470	0.291	0.420	0.089	0.521	0.111	<0.001
Interaction		0.554	0.923	0.065	0.628	0.243	0.227	0.192

Values are represented as means ± SEM (*n* = 8).

a,b Mean values without common superscript differ significantly (*P* < 0.05).

### Lipid Peroxidation and Antioxidant Parameters in the Jejunal Mucosa

**Figure 2:**
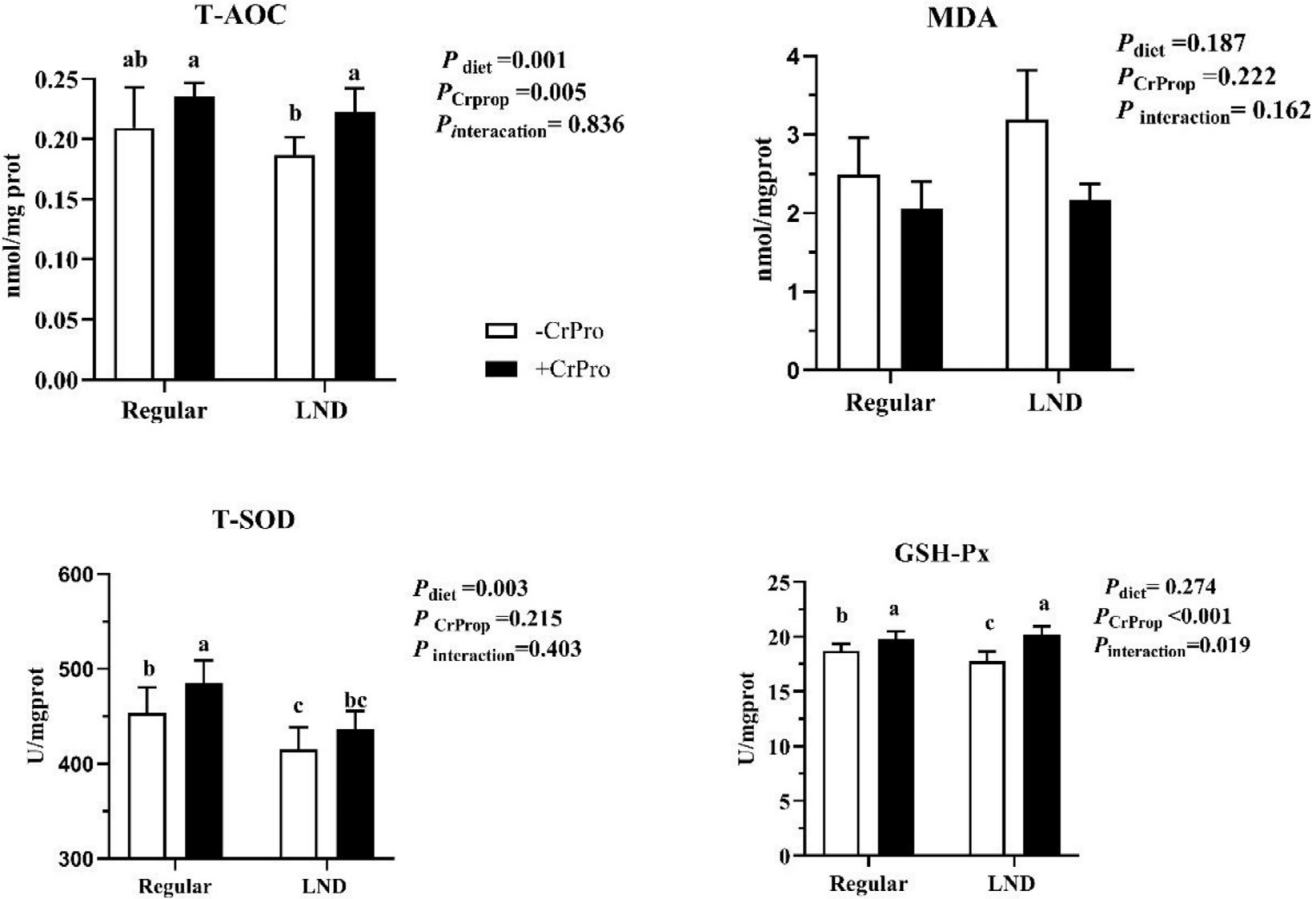
Oxidation and antioxidant parameters respond to dietary nutrient density and CrProp. Values are represented as means ± SEM (*n* = 8). ^a,b^Mean values without common superscript differ significantly (*P* < 0.05).

The LND diet lowered T-AOC levels and T-SOD activity in the jejunal mucosa (Figure 2, *P* < 0.01). Supplementation of CrProp increased T-AOC levels (*P* = 0.005) and enhanced GSH-Px activity compared with groups without CrProp (*P* < 0.01). The interaction between CrProp and regular diet, and CrProp and LND diet, enhanced GSH-Px activity (*P* = 0.019). There was no treatment effect on jejunal mucosa MDA level (*P* > 0.05).

### Gene Expressions in the Jejunal and Hepatic Tissues

**Figure 3:**
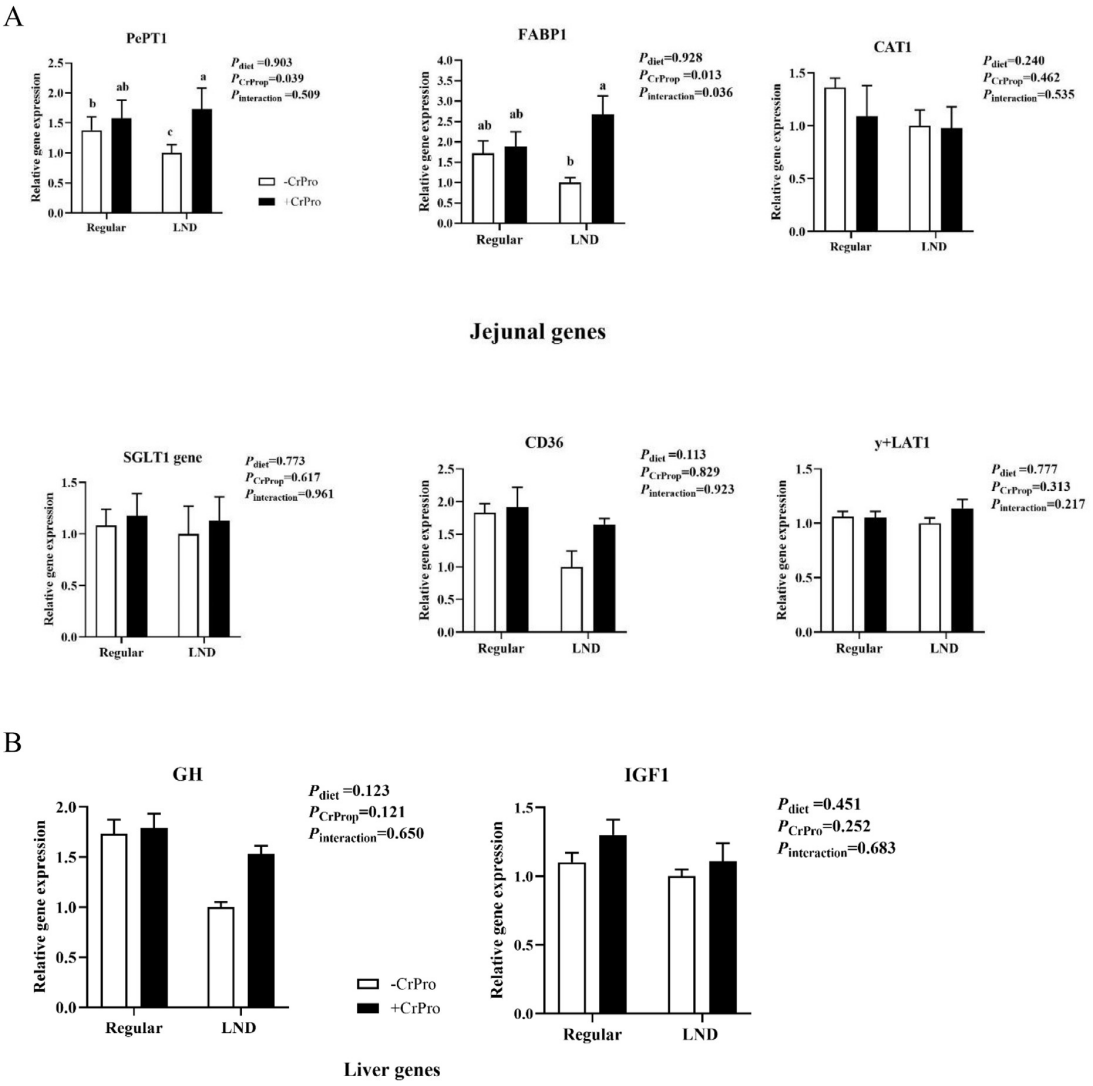
Gene expressions in the jejunum (A) and liver (B) tissues respond to dietary nutrient density and CrProp. Values are represented as means ± SEM (*n* = 6). ^a,b^Mean values without common superscript differ significantly (*P* < 0.05).

There was no treatment effect on jejunal CAT1, SGLT1, CD36, or y+LAT1 gene expressions (Figure 3A; *P* > 0.05). Jejunal FABP1 and PePt1 expressions were increased (*P* < 0.05) after CrProp intervention over the 56 d. The interactive effects of CrProp and LND diet upregulated jejunal FABP1 expression (*P* = 0.036). While GH and IGF1 genes in hepatic tissues were not expressed differently (*P* > 0.05) across treatments, as displayed in Figure 3B.

### Meat Quality

**Figure 4:**
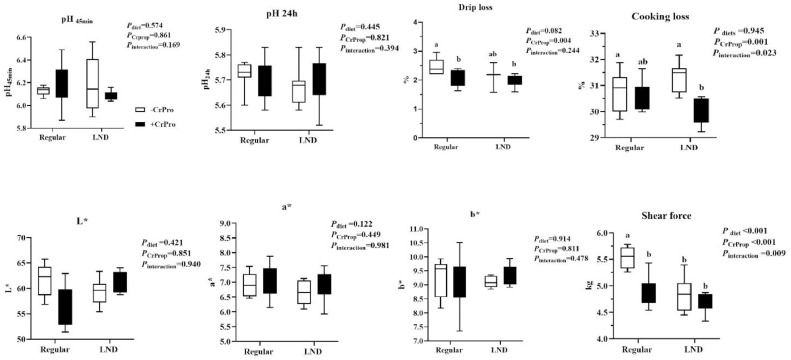
Meat quality at d 56 responds to dietary nutrient density and CrProp. Values are represented as means ± SEM (*n* = 8). ^a,b^Mean values without common superscript differ significantly (*P* < 0.05).

Nutrient density, CrProp, and their interaction did not change muscle pH and meat color (*P* > 0.05) in Figure 4. The LND diet reduced muscle shear force (*P* < 0.01). CrProp supplemented in the diet decreased drip loss, cooking loss, and shear force (*P* < 0.05). The interactive effect between CrProp and regular diet reduced muscle shear force (*P* = 0.009), and it lowered muscle cooking loss of broilers (*P* = 0.023) in CrProp and LND diet group.

## DISCUSSION

### Gut Mucosa Antioxidant Capability

Broilers' antioxidant capacity weakens over time due to chronic stresses, such as changed temperature, epidemic prevention, and BW checks (McKee and Harrison, 1995; Del Vesco et al., 2017; Çetin and Güçlü, 2020). As the first internal barrier, gut mucosa is directly exposed to feed and non-nutritional substances. This leads to the production of excess reactive oxygen and reactive nitrogen species, obstructing nutrient digestion and absorption (Mishra and Jha, 2019). Gut mucosa is vulnerable to oxidation due to a heavy workload while is also protected by an antioxidant defense system, including T-AOC, T-SOD, and GSH-Px. We found that the LND diet lowered T-AOC and T-SOD activity, which agreed with the reports of Attia et al. (2022) and Paraskeuas et al. (2016), where LND diet or low-ME lowered the T-AOC in broilers’ serum. T-AOC reflects the overall antioxidant ability of antioxidant substances and antioxidant enzymes. Through T-SOD, O_2_^−^, and H_2_O are catalyzed to produce H_2_O_2_, which is further decomposed into H_2_O and O_2_ by catalase (Tan et al., 2018). We speculated that an unbalanced-density diet promoted broilers to eat more feed, producing free radicals, and some mycotoxins in the feed were also stressors (Mishra and Jha, 2019). The results from the study suggest that the LND diet may harm the gut.

In the present study, CrProp led to higher GSH-Px activity that eliminates peroxides and hydroxyl radicals, and higher T-AOC levels. Chromium, as a trace element, at a low dose activates the antioxidant system in laying hens under heat stress and in fish (Untea et al., 2019; Chen et al., 2021; Zhang et al., 2022b). Considering CrProp's antioxidant mechanism, Untea et al. (2019) confirmed that adding organic Cr promoted zinc deposition in breast meat. Zinc can protect membranes from iron-initiated lipid oxidation or inhibit the catalytic properties of redox-active transition metals, such as Fe and Cu (Zago and Oteiza, 2001). Sahin et al. (2003) illustrated that dietary Cr^3+^ improved blood vitamin C and E levels (antioxidants) in broilers. On the other hand, CrProp reduced serum corticosterone in this study, a biomarker of oxidative stress (Lapointe et al., 2016), corresponding to enhanced antioxidant activity. As described by Anderson et al. (1991), corticosterone levels declined in humans after Cr regulation. Moreover, supplementation with CrProp improved GSH-Px activity, especially for broilers fed the LND diet, reflecting the strong ability to protect tissue from oxidation.

### Growth Performance

Normal nutrient density diet contributed to heavier BW and more breast yield than low-nutrient density diet with an inconstant ME: CP ratio (reduction of 239 kcal/kg ME and 1.8% CP) in commercial broilers (Zhang et al., 2022a). Nevertheless, we found that BW and ADG had no response to the LND diet with changed ME: CP ratios. According to Namroud et al. (2010), CP below 19% limited broiler growth from 10 to 28 d of age. A reduction in CP by 1 to 2% was detrimental to the overall growth of yellow-feathered broilers (Wang et al., 2022). As CP and AA levels in the LND diet met the standard requirement for the whole period, a reduction of 0.43% CP might not affect the BW and ADG of yellow-feathered broilers. In addition to their premier energy needs for body metabolism, young animals need energy for growth. When broilers were fed low energy diets, their higher feed intake was regulated by the central nervous system and peripheral tissue (Kuenzel et al., 1999), namely the biological features of “eat for energy” (Meloche et al., 2018). The above statements may explain why broilers who fed the LND diet had comparable BW and ADG to those who consumed the regular diet. Low nutrient density diets resulted in a negative influence on the FCR of commercial broilers (Corduk et al., 2007; Niu et al., 2009; Paraskeuas et al., 2016; Attia et al., 2021), which was in agreement with our results on yellow-feathered broilers in the current study.

On the other hand, more feed intake might be a burden for the gut because the digestion and absorption processes produce free radicals (Mishra and Jha, 2019). This reason may explain the weakened intestinal antioxidant activities in LND-treated broilers.

Furthermore, diets high in ME or CP levels promote fat deposition in the abdomen in poultry (Fan et al., 2008; Wang et al., 2013; Attia et al., 2021). In this study, more LND diet consumption by broilers from 1 to 42 d may compensate for the negative impact of energy deficiency on fat deposition. Thus there was no difference in abdominal fat content. Some studies also revealed that carcass weight and breast yield positively correlated with the live BW of broilers (Nahashon et al., 2005; Zhang et al., 2022a). In our study, nutrition levels did not affect BW, corresponding to no carcass trait differences.

Chromium involves in the metabolism of carbohydrates, lipids, and proteins by forming IGF to enhance insulin action. Insulin accelerates glucose clearance and lipid metabolism (Kroliczewska et al., 2004). In livestock, the impacts of Cr^3+^ on growth performance differ. Supplementing 0.5 mg/kg Cr yeast or 0.4 and 0.8 mg/kg CrProp improved ADG and FCR and reduced serum glucose levels in commercial broilers (Kroliczewska et al., 2004; Arif et al., 2019; Fraz et al., 2023). However, other studies with various doses of 0.2 to 1.6 mg/kg CrProp, Cr-yeast, and CrPic showed no effects on BW, ADG, or FCR of broilers (Suksombat and Kanchanatawee, 2005; Jackson et al., 2008; Rama Rao et al., 2012; Zheng et al., 2016; Han et al., 2021). In this study, mortality showed that Cr^3+^ as CrProp at 0.2 mg/kg did not impair health. CrProp improved BW, ADG, and FCR but reduced ADFI during 1 to 21 d. It is mostly possible that Cr promoted insulin sensitivity and glucose absorption in chicken muscle, liver, and adipose tissue (Brooks et al., 2016). In vitro, CrPic promoted insulin binding with pig's red blood cells and fat cells (Matthews et al., 2003). For in vivo studies, 20 of the 46 experiments showed Cr^3+^ effect on declining fasting blood glucose (Molz et al., 2021). However, most data revealed that Cr^3+^ did not affect insulin levels (Han et al., 2021), which was consistent with our results. Moreover, in the current findings, CrProp failed to modulate the expressions of growth hormone (**GH**), which activates GH receptors, or insulin-like growth factor 1 (**IGF1**), which has a high anabolic effect. This cause partially due to the long blood collection interval after fasting (6 h). In addition, we only collected blood on d 56, not at the starter and grower periods, and we did not compare insulin activity and glucose levels before and after eating. Brooks et al. (2016) revealed that glycogen in the liver and muscle tended to increase after fasting and refeeding by feeding a Cr fortified diet to chicks. In this case, we cannot conclude the relationship between CrProp and insulin from this aspect. FABP1 and Pept1 genes were upregulated by CrPro supplementation, which may promote nutrient absorption. Hayat et al. (2020) reported that 0.15 and 0.2 mg/kg Cr promoted CAT1 and GLUT2 gene expressions in broiler jejunal tissues. The upregulated gene expressions may be due to the enhanced gut antistress ability, which may help to explain the improved FCR by CrProp supplementation. The enhanced gut mucosa antioxidant activities protect the gut from being attacked by free radicals and toxins in feed. Moreover, CrProp supplementation reduced ADFI and improved the FCR of broilers in LND and regular groups during 1 to 21 d. CrProp supplementation could compensate for feed conversion loss caused by low-density content and reduce feed cost, which may be a result of enhanced GSH-Px activity and upregulated FABP1 in the jejunum.

Abdominal fat can be considered the total body fat content, most of which is synthesized in the liver (Chen et al., 2018). Abdominal fat is not desirable for consumers hence a waste of energy. As reported earlier, organic Cr compounds decreased abdominal fat deposition in broilers, perirenal fat in fattening lambs, and backfat in finishing pigs (Suksombat and Kanchanatawee, 2005; Jackson et al., 2009; Rama Rao et al., 2012; Moreno-Camarena et al., 2015; Huang et al., 2016; Chen et al., 2018). Cr^3+^ downregulated the expression of the acetyl CoA carboxylase1 gene in goat kidneys, which catalyzes acetyl-CoA to produce malonyl-CoA substrate for fatty acids synthesis and decreases liver lipoprotein lipase activity for fat deposition in broilers (Najafpanah et al., 2014; Chen et al., 2018). It may be an explanation for downregulated fat deposition by CrProp.

### Meat Quality

Yellow-feathered broilers are famed for superior flavor, tenderness, and gustatory experience. There is limited study on the relationship between nutrient levels and CrProp supplementation on the meat traits of this broiler. Earlier data indicated that nutrient density could affect meat quality. Low nutrient density diets reduced shear force in broilers (Zhu et al., 2012; Zhang et al., 2022a), which agrees with our findings. There were reports that shear force in pork and broiler breast meat was reduced as dietary ME increased or CP level reduced (Tang et al., 2007; Zeng et al., 2012; Wang et al., 2013), due to the altered muscle fiber type, size, and density by different diets (Zhao et al., 2012). Reduced shear force indirectly reflected increased tenderness (Tang et al., 2007; Niu et al., 2009). Supplementation of CrProp reduced shear force, drip loss, and cooking loss in this study. It was proved that Cr^3+^ at 0.4 or 0.2 mg/kg decreased the cooking loss of breast meat (Huang et al., 2016; Saracila et al., 2022) and 0.2 mg/kg Cr^3+^ led to higher water holding capacity (Amatya et al., 2004). Interestingly, both main effects of nutrient density and CrProp showed the synergy effect in lowering shear force in this study. Drip loss and cooking loss are water loss in muscle fibers controlled by cell integrity (Okeudo and Moss, 2005), and are highly linked to muscle oxidation (Tang et al., 2021). It was reported that Cr reduced lipid peroxidation and improved SOD and GSH-Px activity in breast meat (Jin et al., 2018; Untea et al., 2019). Although we did not assay antioxidation activity in breast meat, CrProp induced higher T-AOC and GSH-Px activity in the jejunal mucosa. It is possible that Cr administration may protect myofibrils and lipids from oxidation, maintaining cell integrity, which may help explain the reduced water loss after cooking when CrProp was supplemented. Dietary nutrient level and Cr supplementation failed to affect meat color in the present study. It is possible that yellow-feathered broilers contain more red myofibers than white-feathered broilers, making them less sensitive to suboptimal nutrient density (Roy et al., 2006).

## CONCLUSIONS

In this study, yellow-feathered broilers fed low-nutrient density diet ate more and exhibited bigger FCR to maintain similar BW and ADG to broilers consuming the regular diet, and had lowered antioxidant capacity (lower T-AOC and T-SOD). Dietary supplementation of 200 mg/kg CrProp increased BW, ADG, and improved FCR, possibly through enhanced intestinal antioxidant ability (T-AOC and GSH-Px) and upregulated FABP1, GLUT2, y^+^ LAT, PePt1 gene expression. Dietary CrProp improved meat quality by decreasing cooking loss, drip loss, and shear force. Interestingly, this study found that CrProp administration to LND diets improved meat quality (reduced cooking loss), and reduced ADFI but improved FCR in the starter period which may be owing to enhanced jejunal health (higher GSH-Px and FABP1 expression).

## DISCLOSURES

All authors declare that there is no conflict of interest in any person and affairs.
